# Low prevalence of HCV infection with predominance of genotype 4 among HIV patients living in Libreville, Gabon

**DOI:** 10.1371/journal.pone.0190529

**Published:** 2018-01-31

**Authors:** Angélique Ndjoyi-Mbiguino, Arnaud John Kombe Kombe, Berthold Bivigou-Mboumba, Samira Zoa-Assoumou, Falone Larissa Akombi, Francis Nzengui Nzengui, Hervé M’boyis Kamdem, Sandrine François-Souquière

**Affiliations:** 1 Laboratoire National de Référence IST/Sida, Département de Bactériologie-Virologie, Faculté de Médecine et des Sciences de la Santé, Université des Sciences de la Santé, Owendo, Gabon; 2 Unité Mixte de Recherches VIH et Maladies Infectieuses Associées (UMR VIH-MIA), Centre International de Recherches Médicales (CIRMF), Libreville, Gabon; University of Cincinnati College of Medicine, UNITED STATES

## Abstract

**Background:**

Gabon is an endemic area for human immunodeficiency virus (HIV) and hepatitis C virus (HCV) and the risk of co-infection is high.

**Method:**

Between November 2015 and April 2016, we conducted retrospective study on HCV infection among people living with HIV/AIDS (PLHA). A total of 491 PLHA were included in this study and tested for the presence of HCV infection. HIV viral loads were obtained using the Generic HIV viral Load® assay and the CD4+ T cells count was performed using BD FACSCount™ CD4 reagents. HCV screening was performed using the MP Diagnostics HCV ELISA 4.0 kit. HCV genotypes were determined by sequence analysis of NS5B and Core regions. The Mann-Whitney test was used to compare the groups. Chi-2 test and Fisher's Exact Test were used to compare prevalence.

**Results:**

HCV seroprevalence was 2.9% (14/491), (95% confidence interval (CI):1.4–4.3%). The percentage of HCV viremic patients, defined by the detection of HCV RNA in plasma, was 57% (8/14), representing 1.6% of the total population. HCV seroprevalence and replicative infection were not statistically differ with gender. The percentage of co-infection increased with age. No correlation with CD4+ T cells count and HIV viral load level was registered in this study. Identified HCV strains were predominantly of genotype 4 (87.5%) including 4k, 4e, 4g, 4p, 4f and 4c subtypes. Only one strain belonged to genotype 2 (subtype 2q). Analysis of the NS5B region did not reveal the presence of resistance-associated substitutions for sofosbuvir.

**Conclusion:**

A systematic screening of hepatitis C is therefore strongly recommended as well as genotyping of HCV strains in order to adapt treatments for the specific case of people living with HIV/AIDS in Central Africa.

## Introduction

People living with human immunodeficiency virus (HIV) often become infected by hepatitis viruses, whether hepatitis C (HCV), and/or hepatitis B (HBV), occasionally associated with hepatitis Delta (HDV). In addition, reactivation of hepatitis virus in patients infected with HIV is now considered significant [1,2]. These co-infected people are at increased risk for serious, life-threatening complications [3].

HCV infection alone is a leading cause of chronic liver disease in the world. The World Health Organization (WHO) estimated that in 2015 about 170 million people were infected by HCV. In sub-Saharan Africa, the overall HCV prevalence is 5.7% with 32 million people affected, and therefore this region is considered as highly endemic [4]. There were approximately 36.7 (34.0–39.8) million people living with HIV at the end of 2015. Sub-Saharan Africa is the most affected region, with 25.6 (23.1–28.5) million people living with HIV. Based on this figure, about 1 out of 190, and 1 out of 41 people are living with HIV globally and in Africa, respectively [5]. Therefore, all conditions exist to promote co-infections in this part of the world. The HIV/HCV co-infection is associated with an elevated level of HCV viral load and complications in treatment efficacy involved in the fight against HCV [6]. This also affects the rate of transmission of HCV, a factor further aggravating the epidemiological situation in this region of the world. In fact, these co-infected people usually present severe liver fibrosis, with a risk 2 to 5 times higher of developing faster cirrhosis than mono-infected patients [7].

Modes of transmission of HCV are related to blood contact: reuse of needles (poor medical procedures, drug use), blood transfusions, tattoos, piercing, and transplantations. Sexual transmission is less often implicated, excepted in cases of men having sex with men. Finally, the vertical transmission rate is low, around 5%, but it increases by 2 to 5 times in cases of HIV-HCV co-infection [8].

The HCV viruses are classified into 7 genotypes, each devised in subtypes (a, b, c…). HCV genotype 1a is most commonly detected in Europe and the USA, while genotype 1b is distributed worldwide with high prevalence in Europe and the USA as well as in Japan. Genotype 2 is common in Europe, Japan, North America and in West Africa. Genotype 3 is most common in India, Indonesia and South-East Asia. Genotype 4 appears to be prevalent in Central Africa and the Middle East, while genotype 5 is most frequently reported in South Africa. Genotype 6 is found in Hong Kong and South-East Asia [9]. Recently, the last HCV-7 genotype was identified in four patients originating from the Democratic Republic of Congo [10].

In Central Africa, several studies report that HCV prevalence varies widely between countries, ranging from 0.6% to 50.6%, depending on the populations studied, the age and the transmission routes [11]. On average, HCV seroprevalences were 1.1% (95% CI:1–1.3%) in Cameroon [12]; 3.7%, (95% CI: 3.0–4.4) in Equatorial Guinea [13]; 4.7% (95% CI: 2.9–6.5) in the Central African Republic (CAR) [14]; 5.6% (95% CI 4.0–7.2%) in the Republic of the Congo [15] and 2.9% (95% CI 1.5–4.3%) in the Democratic Republic of Congo (DRC) [16].

In Gabon, HCV prevalence varies from 2.1% among pregnant women in urban areas, to 11.2% in rural areas, and can reach 20.4% in very remote villages [17–19]. Prevalence generally increases significantly with age, reaching 62% in people 65 years. The majority of strains belongs to genotype 4, with 4e (majority), 4c, 4p and 4r. The HCV genotypes 1 and 2 were also found in 5.7% and 2.2% of cases respectively [17–19]. The subtype 4e is the oldest in this region of the world and its spread probably has iatrogenic origin [20].

A recent study performed in Gabon, to provide comprehensive guidance for HCV testing, indicated that the HCV prevalence was 7.3% among patients living with HIV in semi-rural areas [21].

Gabonese guidelines on HIV/AIDS recommend hepatitis C screening. Recently, oral direct-acting antiviral (DAA) combination regimen of sofosbivir (SOF) and daclastavir (DCV) is affordable.

In addition, the development of new technical platforms in Gabon allows the virological follow-up of the treated patients.

In this context, it is important to know the HCV prevalence among people living with HIV/AIDS (PLHA) in order to establish future needs. For this, we propose to determine the prevalence of HCV in PLHA and to describe the diversity of HCV strains circulating in Libreville, capital of Gabon.

## Material and methods

### Patients

This retrospective study was carried out from November 2015 to April 2016 in Libreville, Gabon. HCV antibody detection was performed on 500 blood samples taken from PLHA who came to the laboratory as part of their virological and immunological follow-up (HIV viral load and CD4+ T cells count). For 9 of them, the data were insufficient and were excluded from the study. For 491 patients, the data on gender, age and antiretroviral treatment were provided by clinicians. The study was approved by the Ethics Committee of Gabon (N°0021/2013/SG/CNE). Written informed consent was obtained from each patient.

### Sampling and HCV serology

Whole blood specimens were collected in EDTA-containing tubes. After centrifugation at 800g, 10 min, resulting plasma was frozen at -80°C. All plasma samples were tested for HCV IgG antibodies using the third generation ELISA test (MP Diagnostic HCV ELISA 4.0, MP Biomedicals, Singapore 627885) based on double antigen “sandwich” principle and according to the manufacturer’s instructions. Specimens with a signal-to-cutoff S/CO ratio <0.7 were considered non-reactive, and those with an S/CO ratio ≥1.0 were considered reactive. All specimens with an S/CO ratio ≥1.0 including one specimen with S/CO ratio between 0.7 and 1.0 were further tested with an immunochromatographic assay (HCV Serum Cassette, MEDIFF, Aubagne, France) for confirmation. The specimen with S/CO ratio between 0.7 and 1.0 was non-reactive and was considered as negative.

### HIV-1 RNA viral load

Viral RNA extractions were performed from clarified plasma, (centrifugation at 3000g/10 min) using Arrow Viral NA™ in NorDiag Arrow automatic extraction system (DiaSorin, Dublin, UK). HIV-VLs were obtained using the Generic HIV viral Load® assay with a detection limit of 100 copies/mL (Biocentric, Bandol, France).

### CD4+ T cells count

The CD4+ T cells count was performed using a BD FACSCount™ cytometer with a total of 50 μl of whole blood using BD FACSCount™ CD4 reagents (BD Biosciences France, Le Pont de Claix, France) according the recommendations of the manufacturer.

### HCV sequencing and genotyping

Specimens positive for HCV antibodies were submitted for HCV genotyping. RNA was extracted from 140 μL of clarified plasma (after centrifugation at 700 *g* for 10 min) with the QIAamp RNA viral Mini Kit (Qiagen, Courtaboeuf, France), as recommended by the manufacturer. 10μl of RNA were used to perform both RT-PCRs.

To know the prevalence of patients with active infection, avoiding constraints on the estimate imposed by variability in HCV, we first conducted amplifications of HCV RNA in the most conserved 5 'UTR region [22]. In a second step, to determine the HCV genotype, RNA was amplified targeting two other regions (NS5B and Core) [23].

Thus, for the gene encoding NS5B region, amplifications of fragment of 358bp were carried out with Pr3/Pr4 primer pairs for RT-PCR, and Pr3/Pr5 for the semi-nested PCR. Similarly, fragments of 384 bp of the gene region encoding the Core protein were amplified with CoreOS/CoreOAS primer pairs for RT-PCR, and with CoreIS/CoreIA for the nested PCR as described previously in Njouom et *al* [20].

Sequencing was performed by Macrogen Europe (Amsterdam, the Netherlands). Pairwise alignments were performed using MEGA 6.06 software [24] that generated neighbour-joining trees using a Kimura two-parameters model [25]. The reference sequences representing all HCV genotypes were retrieved from the GenBank (https://www.ncbi.nlm.nih.gov/nucleotide/) and from Hepatitis C Virus (HCV) Database Project (https://hcv.lanl.gov/content/index). The phylogenetic trees were visualized by FigTree version 1.4.2 (program 2006–2014, Andrew Rambaut Institute of Evolutionary Biology, University of Edinburgh. http://tree.bio.ed.ac.uk/). All the nucleotide sequences were submitted to GenBank database under the accession numbers KY661736 to KY661751.

### Statistical analysis

Continuous data were expressed as median value with interquartile ranges (IQR) and comparisons between groups were conducted using the Mann-Whitney test with Statistica v.7.1. software (StatSoft France, 2005; www.statsoft.fr). Chi-2 test and Fisher's Exact Test were used to compare prevalence. *p* values < 0.05 were considered to be statistically significant.

## Results

### Characteristic of the population studied

Out of the 491 people living with HIV/AIDS (PLHA) enrolled in this study, a large majority (66.6%) were female. The median age of the population was 44years (interquartile range [IQR]: 37–52) with no significant difference between females (42 years [IQR: 36–50]) and males (48 years [IQR: 43–55]). Among them, 72.9% (n *=* 358; 95% CI: 68.9–76.8%) received antiretroviral therapy and, 66.5% (n = 238; 95% CI: 61.5–71.3%) of these were female. The median CD4+ T cells count in the population studied was 256 cells/μL (IQR: 100–406) and that of HIV-VL was 666 copies/mL of plasma (IQR: 99–230500) (S1 Table).

### Prevalence of HCV in HIV patients in Libreville

In our study, antibodies against HCV were found in 2.9% of PLHA (n = 14; 95% CI: 1.4–4.3%). Out of these, the HCV RNA was detected in 57.1% (8/14), 1.6% of the total population (95% CI: 0.5–2.7%) (Table 1).

**Table 1:** Seroprevalence and HCV RNA by gender, age group, ART treatment, CD4+ T cells and HIV-VL groups.

		HCV serology		HCV RNA	
		Pos % (n)	p value	Pos % (n)	p value
**Total**		**2.9 (14/491)**		**1.6 (8/491)**	
**Gender**	**Male**	**4.3 (7/164)**	**p = 0.25**	**2.4 (4/164)**	**p = 0.45**
	**Female**	**2.1 (7/327)**		**1.2 (4/327)**	
**Age group**	**18–29**	**0.0 (0/34)**	**p = 0.21**	**0.0 (0)**	**p = 0.06**
	**30–59**	**2.8 (11/398)**		**1.3 (5)**	
	**≥ 60**	**6.5 (3/46)**		**6.5 (3)**	
**ART treatment**	**Yes**	**2.3 (9/398)**	**p *=* 0.15**	**1.5 (6/398)**	**p = 0.65**
	**No**	**5.3 (5/93)**		**2.2 (2/93)**	
**CD4+ T cells count** **(cells/**μ**L)**	**≤200**	**2.6 (5/191)**	**p = 0.59**	**1.0 (2/191)**	**p = 0.37**
	**200–350**	**2.1 (2/95)**		**2.1 (2/95)**	
	**351–500**	**2.4 (2/84)**		**1.1 (1/84)**	
	**CD4˃500**	**5.3 (4/75)**		**4.0 (3/75)**	
**HIV-VL (copies/mL)**	**≤10^2^**	**3.1 (7/223)**	**p = 0.99**	**2.2 (5/223)**	**p = 0.91**
	**10^2^–10**^**4**^	**1.7 (1/60)**		**0,0 (0/60)**	
	**10**^**4**^**–10**^**6**^	**3.1 (4/128)**		**1.7 (2/128)**	
	**>10**^**6**^	**2.6 (2/76)**		**1.3 (1/76)**	

p values have been calculated by Chi2 or Fisher's Exact tests.

The medians of CD4+ T cells and HIV-VL were respectively 350 cells/μL (IQR = 93–561) and 1520 copies/mL (IQR = 99–355000). HCV prevalence was 2.1% (n = 7; 95% CI: 0.5–3.7%) in female and 4.3% (n = 7; 95% CI: 1.2–7.4%) in male. HCV viral RNA was detected in 1.2% of female and 2.4% of male, respectively. However, HCV seroprevalence and replicative infection were not statistically differ with gender (p = 0.25 and p = 0.45). Serological and molecular prevalence were higher in older patients but without statistical significance (p = 0.21 and p = 0.06). No correlation with CD4+ T cells count and HIV viral load level was registered (Table 1). All data collected for 14 PLHA co-infected with HCV are detailed in Table 2. Among these co-infected patients 9 were on ART, including 6 with a 1^st^ line ART treatment. No patient was taking a 3^rd^ line ART treatment (Table 2).

**Table 2:** Characteristics of HIV-HCV co-infected patients.

ID	Age	Sex	ART regimen	CD4^+^ T cells (cells/μL)	HIV-VL (copies/mL)	HCV Genotype
**33245**	**59**	**M**	**No**	**729**	**238 000**	**NA**
**33274**	**52**	**F**	**No**	**70**	**220 000**	**NA**
**33588**	**43**	**M**	**TDF-FTC-EFV**	**124**	**undetectable**	**NA**
**34227**	**44**	**F**	**TDF-3TC-EFV**	**7**	**2 940**	**NA**
**34574**	**44**	**M**	**No**	**NA**	**>5 000 000**	**NA**
**34608**	**50**	**F**	**TDF-FTC-EFV**	**500**	**undetectable**	**NA**
**33301**	**50**	**F**	**TDF-3TC-EFV**	**350**	**undetectable**	**4f**
**33516**	**49**	**F**	**No**	**561**	**>5 000 000**	**4k**
**33926**	**46**	**M**	**ABC-3TC-EFV**	**92**	**684 000**	**4k**
**33994**	**72**	**M**	**TDF-3TC-LVP/r**	**3**	**undetectable**	**2q**
**34565**	**63**	**F**	**No**	**793**	**undetectable**	**4p**
**34610**	**52**	**M**	**AZT-3TC-LPV/r**	**371**	**354 999**	**4g**
**34658**	**41**	**M**	**TDF-FTC-EFV**	**280**	**undetectable**	**4e**
**34667**	**60**	**F**	**TDF-3TC-EFV**	**1385**	**undetectable**	**4c**

NA: ARN HCV none amplified by RT-PCR. No: untreated; AZT: zidovudine; ABC: abacavir; TDF: tenofovir; 3TC: lamivudine; FTC: emtricitabine EFV:efavirenz; LPV/r: lopinavir/rotonavir boosted

### Genotype distribution of HCV

**Fig 1:**
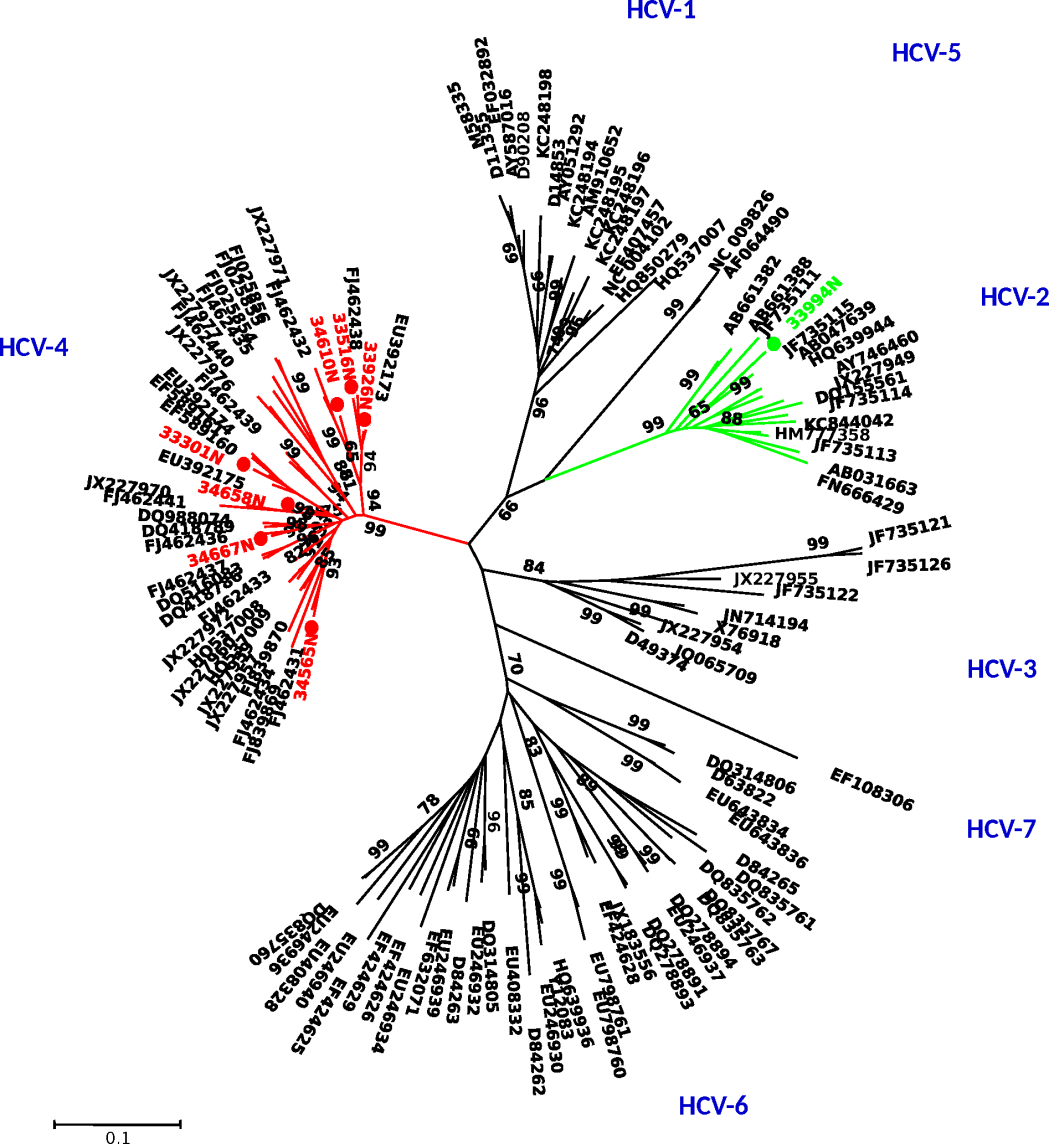
Phylogenetic analysis of HCV NS5B sequences. Neighbor-joining phylogenetic tree constructed with our sequences and reference sequences for HCV genotypes retrieved from the GenBank. The Kimura two-parameter method of estimating genetic distance was used. Numbers next to the nodes of the tree represent bootstrap values (1000 replicates). Branches for genotype 4 are indicated in red and in green for genotype 2. Our sequences are preceded by a spot. The Gen-Bank accession numbers of the new NS5b sequences of HCV are KY661744 to KY661751.

**Fig 2:**
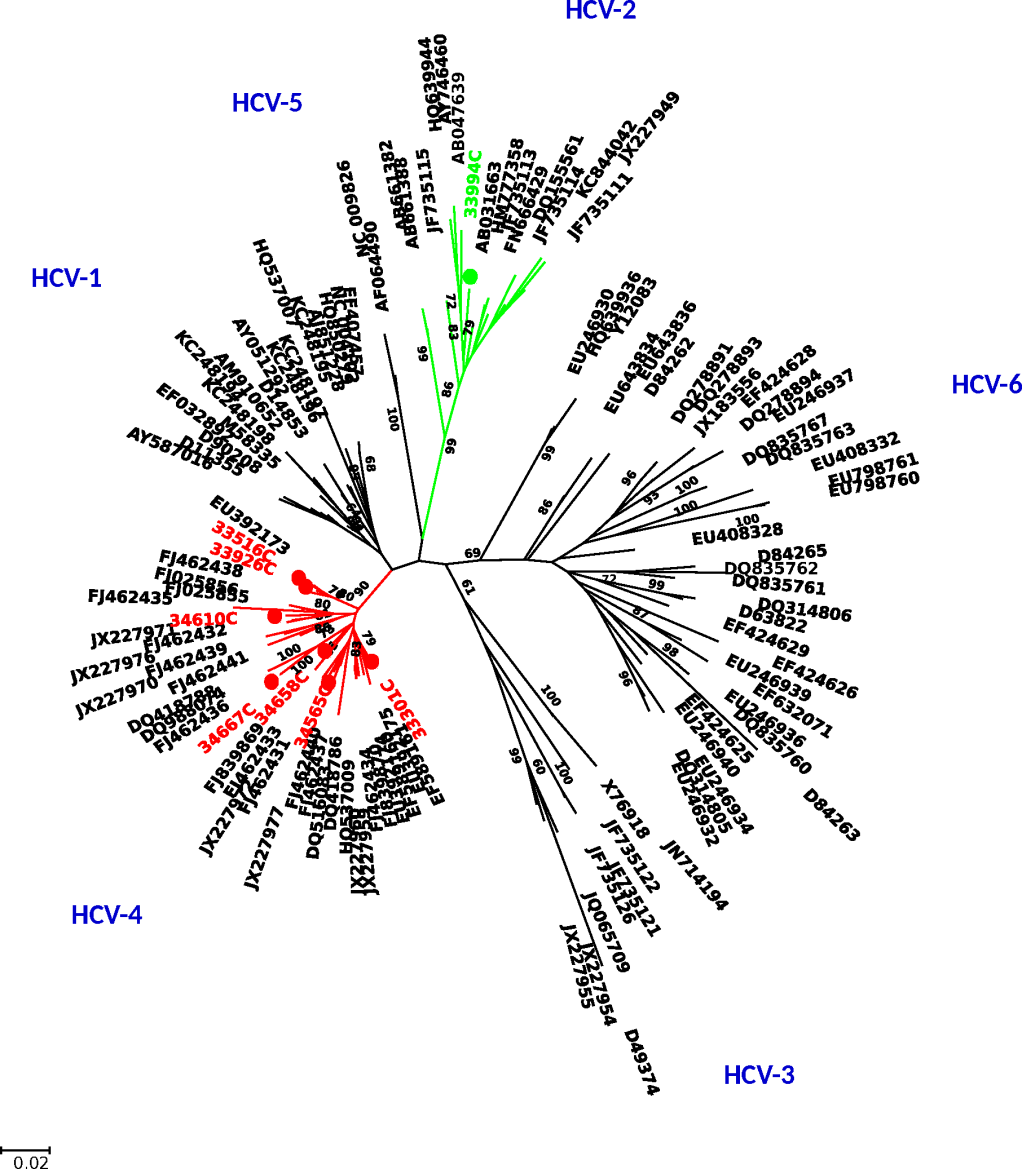
Phylogenetic analysis of HCV Core sequences. Neighbor-joining phylogenetic tree constructed with our sequences and reference sequences for HCV genotypes retrieved from the Gen-Bank. The Kimura two-parameter method of estimating genetic distance was used. Numbers next to the nodes of the tree represent bootstrap values (1000 replicates). Branches for genotype 4 are indicated in red and in green for genotype 2. Our sequences are preceded by a spot. The Gen-Bank accession numbers of the new core sequences of HCV are KY661736 to KY661743.

Out of the fourteen HCV seropositive patients, we successfully amplified HCV RNA in 8 patients for the three regions studied (5'UTR, NS5B and core). Firstly, to determine the genotype we performed alignment of our sequences with reference sequences of HCV genotypes in core and NS5B regions. As shown in Figs 1 and 2, seven of the HCV strains clustered with genotype 4 and one HCV strain with genotype 2 with absolute consistency in the two regions studied.

In addition, we compared our NS5B sequences with 211 sequences obtained by Njouom *et al*. 2012, including the representative strains from all the provincial capitals of Gabon.

Two our HCV strains were closely related to subtype 4k, one respectively to subtype 4g, 4p, 4f, 4c, and the last one to 4e (S1 Fig). Our only HCV-2 strain was closely related to a strain (JN 642974) from northeastern Gabon belonging to the subtype 2q (S2 Fig). Finally, analysis of the NS5B region did not reveal the presence of the L159F, S282T and V321A mutations, the major resistance-associated substitutions for sofosbuvir.

## Discussion

This study was conducted to assess the impact of HCV co-infection in PLHA. Through this study, we recorded a low HCV prevalence (2.9%). This prevalence increased significantly with age but without statistical significance with regard to gender (p = 0.25). Our result in PLHA is similar to those described previously in pregnant women, from Libreville, with an average prevalence of 2.1% (1.3% among 14–20 years old and 6% among those over 35 years old) [26]. Conversely, in remote rural areas in Gabon, HCV prevalence was higher, about 20.7% and over 62.2% in people over 65 years old [17]. In other rural areas of the estuary (near Libreville), seroprevalence was 12.7% (up to 30.5% in people over 55 years old) [20]. Several studies have suggested that the spread of HCV in these high HCV endemic rural areas could not be attributable to intra-family or sexual transmissions, but rather to a cohort effect, with events in the last century, occurring iatrogenic events [19,20,26].

Recently, a study conducted in PLHA from Franceville (Haut-Ogooué province) showed an average prevalence of 7.3% [21]. This prevalence is 3 times greater than that obtained in our study. In these two studies (semi-urban versus urban), 2/3 of the patients received ART (66.5% versus 76%) and the average age (42.3 versus 43.7) and sex ratio (1.4 versus 1.8) were comparable. The observed difference is probably related to the types of populations included (semi-urban areas versus urban). Indeed, as already demonstrated in the general population, the inhabitants of rural areas, forming a closed cohort, are more often infected with HCV than people in urban areas [20].

A similar trend was found in Cameroun, where PLHA living in cities were less likely to be infected with HCV compared to those in rural villages although the difference was not statistically significant [27].HCV spread in Central Africa was arguably by iatrogenic routes during past campaigns for the control of epidemic tropical diseases in the last century. [19,28,29]. Main risk factors for continual circulation of HCV may be unsafe injections, improperly sterilized medical equipment, and ritual behavior [26]. But the spread of HCV may also be influenced by social and economic status between urban and rural settings [30]. Our study does not explain this difference with scientific arguments. However, we can assume a better access to medical care in Libreville, a higher social level and a decrease in ritual usage. All are in favor of a decline in HCV transmission in urban areas.

Average prevalence of HCV for PLHA in Central Africa was estimated at 5.87% (95% CI: 3.71‒8.04) [4]. In Cameroon, according to the studies, the HCV prevalence in the HIV population varies from 2.8% to 7.2% [27,31]. Our data are consistent with those found at the HIV treatment centers in the South West Region of Cameroon (2.8%) [31].

Out of 14 HCV seropositive patients, 8 were viraemic (57.1%), corresponding to the overall prevalence of 1.6%. This rate of replicative infection corroborates those from previous studies [17,18]. However, the real impact of HIV infection could not bring to light because of the insufficient number of cases of co-infection.

Phylogenetic analyses indicate the circulation of two HCV genotypes (4 and 2) in PLHA in Libreville. Genotype 4 was predominantly found in 7 of 8 (87.5%) HCV viraemic patients, followed by genotype 2 in one patient. These HCV genotypes were previously found in all studies in Gabon [17,20]. The subtypes are very heterogeneous: 4k, 4g, 4p, 4f, 4c and 4e, the latter being the subtype mostly found.

Genotype 2 has already been described, with a prevalence of 2.2% in Gabon, but not in the area of the Estuary [20]. We did not find the HCV genotype 1 during our study. The mode of transmission of HCV is in constant evolution and globalization, immigration, sexual changes and drug trafficking could act as new vectors of HCV dissemination [32]. In fact, the introduction of new genotypes in urban areas by these new routes should not be neglected in the future. This has been demonstrated in numerous studies, such as the introduction of genotype 4 in France (constant increase) and in Italy [33,34]. Finally, in our study, we didn’t find recombinant HCV strain in Gabon, as recently evidenced in southern Cameroon [35].

For the purpose of eradicating HCV, the oral HCV direct-acting antiviral combination with sofosbuvir/daclastavir is available in Gabon. The absence of naturally occurring sofosbuvir resistance-associated variants for both genotypes 4 and 2 is comforting for the choice of this strategy and its long-term effectiveness.

However, there is some limitation to this study. Information on some known risk factors for HCV was missing and should be incorporated into future studies In addition, wider sampling will enable more statistical power in the analysis of the impact of HCV in PLHA. Finally, future studies should incorporate biochemical values of liver function as well as HCV viral load values.

Presently, one of the challenges is the implementation of an epidemiological and molecular surveillance of Gabonese population. Diagnosis of HCV genotypes is clinically relevant as genotypes differ in their response rates to the current standard treatment [36]. Additionally, the recent new drugs against HCV, such as DAA have some interactions with medications prescribed for HIV [37]. Then, in order to provide the support necessary for co-infection HIV/HCV surveillance, it is imperative to offer quality analysis, rapid and inexpensive, performed at a national scale.

## Conclusion

Contrary to expectations, the prevalence of HCV in PLHA is low in Libreville. The risk factors for the acquisition of this infection in urban areas should be sought as they appear to be clearly different to those in rural areas. However, as the circulating genotypes are identical to those previously found in Gabon we assume that the origin of these HCV strains remains local. Finally, systematic screening, quantification of HCV RNA and genotype characterization in PLHA must be integrated to the health system in order to obtain quality care.

## Supporting information

S1 Table Characteristic of all population studied (Age, Sex, CD4^+^ T cells, HIV-VL, Ab HCV, ARN detection in 5'UTR, NS5B and core regions, and genotypes). (XLS)

S1 Fig Phylogenetic analysis of HCV NS5B sequences of genotype 4 with previously reported Gabonese reference strains. **N**eighbor-joining phylogenetic tree constructed using the Kimura two-parameter method of estimating genetic distance. Numbers next to the nodes of the tree represent bootstrap values (1000 replicates). Gabonese strains are in red. Our sequences in bold are preceded by a red spot.
(TIFF)

S2 Fig Phylogenetic analysis of HCV NS5B sequences of genotype 2 with previously reported Gabonese reference strains. Neighbor-joining phylogenetic tree constructed using the Kimura two-parameter method of estimating genetic distance. Numbers next to the nodes of the tree represent bootstrap values (1000 replicates). Gabonese strains are in red. Our sequence in bold is preceded by a green spot.
(TIFF)
