# Supplementary figures and images for: Low prevalence of HCV infection with predominance of genotype 4 among HIV patients living in Libreville, Gabon
Source: PLoS One. 2018 Jan 31;13(1):e0190529. doi: 10.1371/journal.pone.0190529 (PMC5791959; doi:10.1371/journal.pone.0190529)

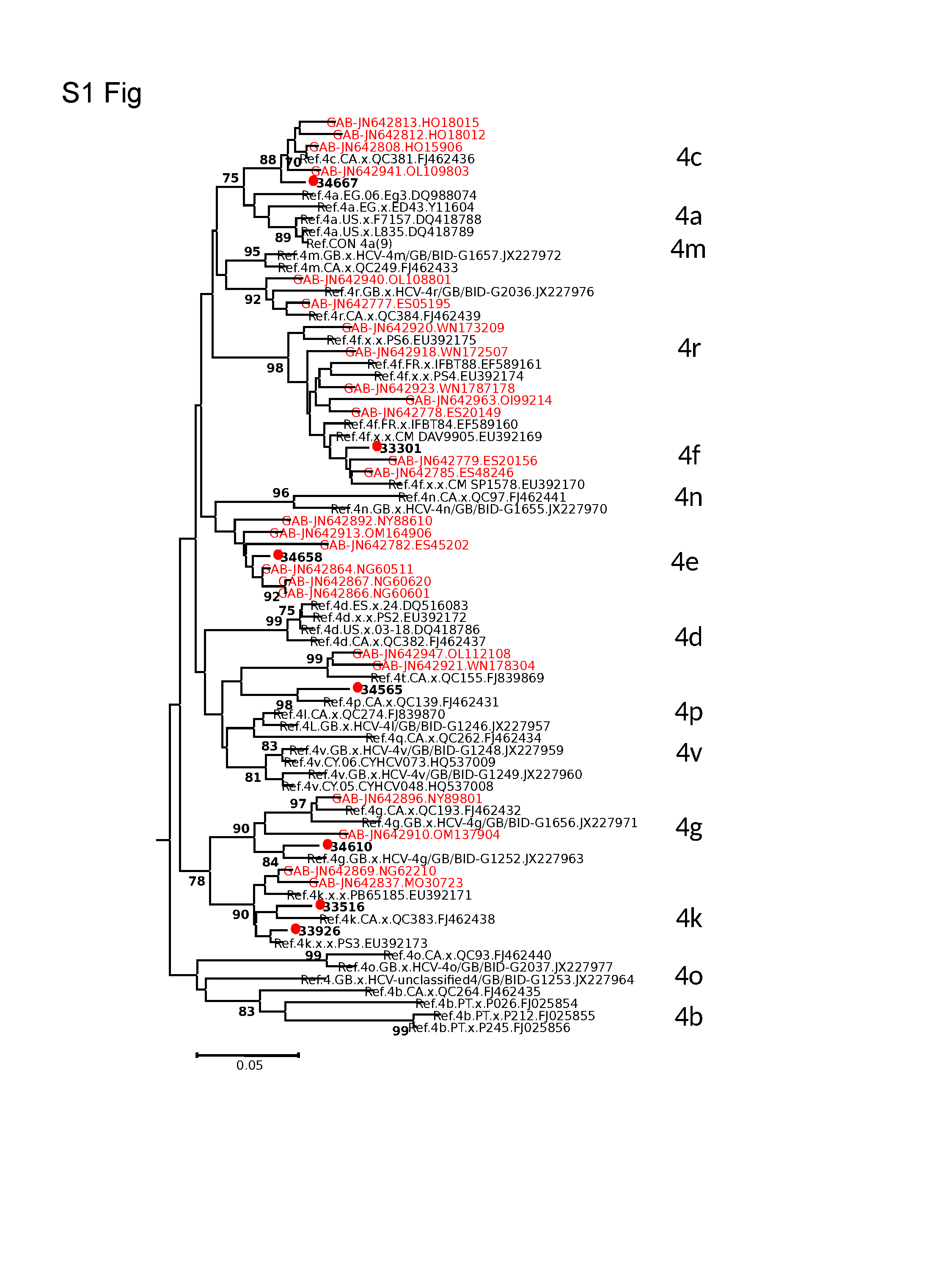

Supplement: S1 Fig — Neighbor-joining phylogenetic tree constructed using the Kimura two-parameter method of estimating genetic distance. Numbers next to the nodes of the tree represent bootstrap values (1000 replicates). Gabonese strains are in red. Our sequences in bold are preceded by a red spot. (TIFF) [file pone.0190529.s002.tiff]

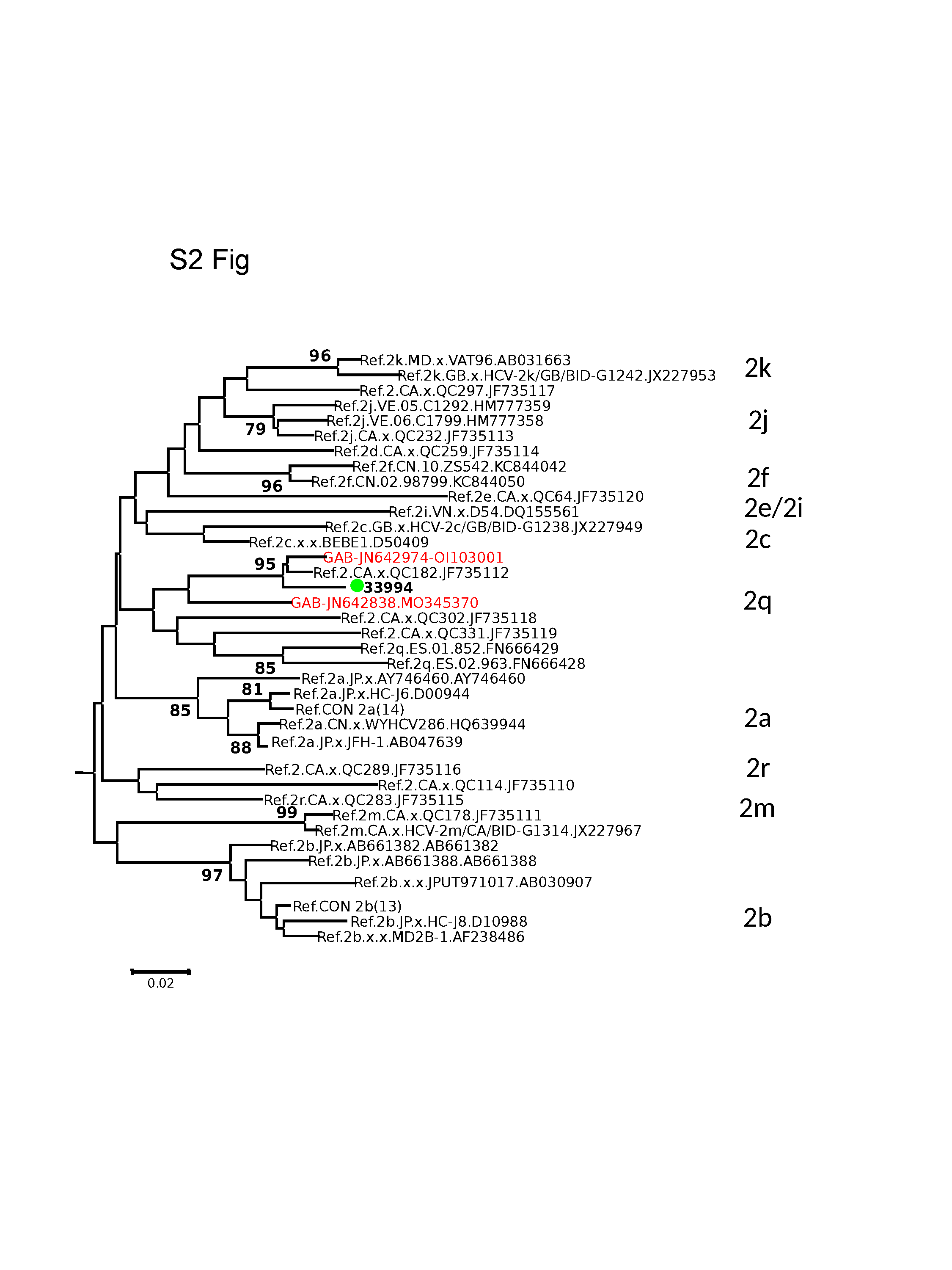

Supplement: S2 Fig — Neighbor-joining phylogenetic tree constructed using the Kimura two-parameter method of estimating genetic distance. Numbers next to the nodes of the tree represent bootstrap values (1000 replicates). Gabonese strains are in red. Our sequence in bold is preceded by a green spot. (TIFF) [file pone.0190529.s003.tiff]
